# Dataset on waste management behaviors of urban citizens in large cities of Indonesia

**DOI:** 10.1016/j.dib.2020.106053

**Published:** 2020-07-22

**Authors:** Agus Brotosusilo, Dwini Handayani

**Affiliations:** a Faculty of Law, Universitas Indonesia, Depok, West Java, Indonesia; b Faculty of Economics and Business, Universitas Indonesia, Depok, West Java, Indonesia

**Keywords:** Urban, Waste management, Indonesia, Regional waste management differences

## Abstract

This study used a statistical approach to measure how urban citizens in certain provinces of Indonesia handle their waste. It illustrates how the desirable habits related to environmental consciousness differ across urban citizens among different regions and economic classes. A wide disparity was found in people's understanding of a healthy and clean environment across provinces and cities. Waste management ignorance was also found to be prevalent. Inculcating personal awareness of the local environment was found to be a good start toward keeping the environment clean. The observed positive correlation between the overall living conditions and littering behavior indicates that households that exhibit littering behavior also tend to score higher on living conditions. This significant positive correlation is indicative of self-interest and ignorance. The study also suggests that a higher level of household economic prosperity correlates with a more desirable behavior toward maintaining a clean and healthy environment; such behaviors are also adopted by citizens living in clean neighborhoods. Furthermore, a clean and healthy lifestyle is also supported by environmental consciousness in conjunction with hygienic environmental conditions.

Specifications Table

**Table:** 

Subject	Environmental Science
Specific subject area	Waste Management Behavior
Type of data	Table
How data were acquired	Data were acquired from a primary data survey across Indonesia's large cities in six provinces. The survey utilized a questionnaire, which is attached as Supplementary File S1. The compiled answers related to the questionnaire are provided as Supplementary File S2.
Data format	Raw Analyzed
Parameters for data collection	The questionnaire was developed to measure urban citizens in certain provinces of Indonesia handle their waste
Description of data collection	The data used in this study were obtained from members of 600 households (respondents) living in 6 different provinces in Indonesia. The data from nearly 100 households were gathered from the cities within each of these provinces.
Data source location	Provinces: Special Region of Jakarta, Jambi, West Sumatra, West Java, East Java, MalukuCities: West Jakarta, East Jakarta, Central Jakarta, North Jakarta, South Jakarta, Jambi, Muaro Jambi, Padang, Surabaya, Tasikmalaya, AmbonCountry: IndonesiaLatitude and longitude: - Special Region of Jakarta: 6.2088° S, 106.8456° E - Jambi: 1.4852° S, 102.4381° E - West Sumatra: 0.7399° S, 100.8000° E - West Java: 7.0909° S, 107.6689° E - East Java: 7.5361° S, 112.2384° E - Maluku: 3.2385° S, 130.1453° E
Data accessibility	With the article

## Value of the Data

• These inferential statistical data and analyses are useful to understand how lifestyle and littering behavior affect the self-assessed living conditions of Indonesia's urban citizens.
• Indonesia's urban citizens as well as policymakers can benefit from these data. The data show that environmental conditions can be improved by enhancing the economic prosperity and focusing on the education of the urban citizens.
• The study data can aid policymakers at the city- and province-level in reshaping citizen behavior regarding waste management.
• The study's analysis of representative cities indicates a degree of ignorance regarding the environment in Indonesia's urban citizens; in these cities, a positive relationship is observed between desirable “environmental habits” and living conditions. Hence, to aid in solving the waste-handling problem, the issue of ignorance must be addressed.
• This analysis also suggests that correlations between certain waste-handling behaviors, socioeconomic factors, and other external factors affect the self-assessed living conditions.

## Data description

**Fig. 1:**
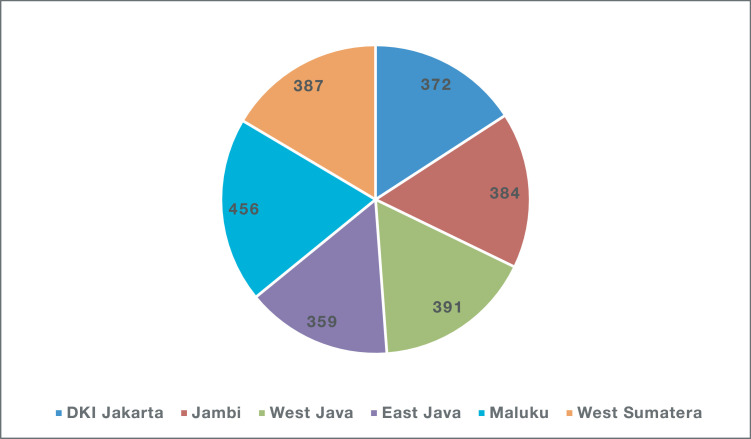
Number of observations in various provinces studied.

**Fig. 2:**
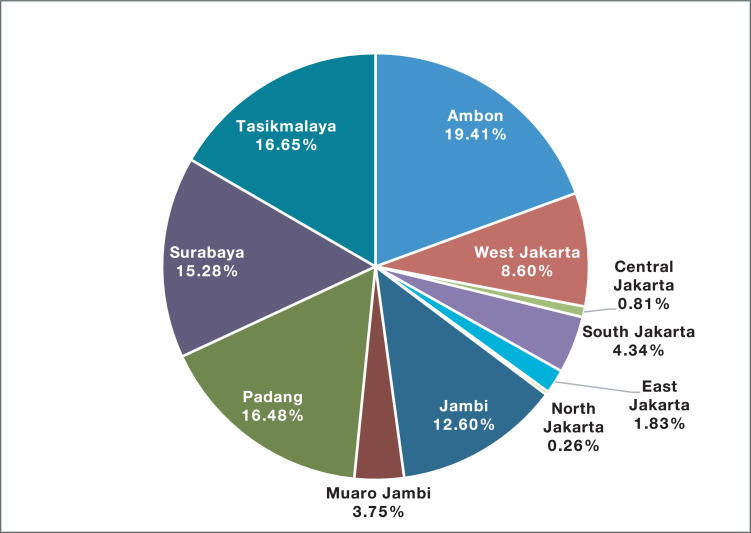
Number of observations in various cities within the study provinces.

The data used in this study were acquired from over 600 households in 6 different provinces in Indonesia. Data were gathered from 100 households in different cities within these provinces. The same family ID was used across all provinces (that is, Family ID 01 exists for each province). However, each family ID was unique within the province; only one family was coded 01 in each province, and no two families were assigned the same code within the same province. The number of observations by provinces and by cities are shown in Fig. 1, Fig. 2, respectively. The complete questionnaire used to gather the data is provided as Supplementary File S1. The questionnaire consisted of seven sets of questions covering the descriptions of spatial and household recognition, household summary, household members, self-regulated garbage disposal routines/habits, knowledge and contribution to waste-handling, perception and attitudes on waste handling, and surveyor-guided self-assessment conditions. The answers to each question in the questionnaire were compiled and are presented in another supplementary file.

The provinces corresponding to these data include the Special Administrative Region of Jakarta, Jambi, West Java, East Java, Maluku, and West Sumatra. Sampled cities include Jakarta, Ambon, Tasikmalaya, Jambi, Muaro Jambi, Padang, and Surabaya. These cities were chosen as they are characterized by relatively high urban populations within the provinces of interest. Meanwhile, provinces were chosen based on their population density in conjunction with the magnitude of the waste-handling problem. The cities were also chosen as representative major cities in Indonesia with urban waste management problems. Here, in the following discussion, the words “respondents” and “urban citizens” are used interchangeably, and both refer to the urban citizens who participated in this research.

In the study, the questions on waste-handling activities were classified into certain categories. These categories cover self-regulated routines/habits with regard to trash disposal, waste disposal facilities, waste-handling awareness and perceptions, knowledge and contribution, and “self-assessment” of lifestyle [1].

Moreover, citizens across the economic strata were chosen for random sampling. The respondents were queried on behavioral information related to waste-handling activities. To ensure greater depth of analysis, the respondents were also asked about their income and expenditure per capita per day. Table 1 lists the general information on the waste-handling behavior of the respondents in each province.

**Table 1:** Distribution of respondents by income and waste-handling behavior.

	Jakarta	Jambi	West Java	East Java	Maluku	West Sumatra	Total
Income per Capita (USD/Day)							
Mean	11.41	11.51	7.40	9.04	19.55	11.15	11.90
Std. Dev.	4.53	6.33	3.40	2.27	13.38	7.67	8.04
Freq.	354	340	117	359	280	376	1826
Expenditure per Capita (USD/Day)							
Mean	8.34	7.59	8.47	7.38	14.22	7.43	9.08
Std. Dev.	3.41	3.74	4.63	2.42	9.35	2.89	5.80
Freq.	355	370	117	355	414	385	1996
How many times a day does (Mr./Mrs.) dispose of trash? (times per day)							
1	26	83	79	34	80	90	392
2	0	9	19	55	11	9	103
3	12	4	2	11	4	0	33
4	0	0	0	0	1	0	1
5	31	2	0	0	0	0	33
6	19	0	0	0	0	0	19
Total	88	98	100	100	96	99	581
Where was the last time (Mr./Mrs.) disposed of trash?							
Trash bin	91	78	101	100	63	61	494
On the street	0	1	1	0	24	0	26
Drainage	0	0	1	0	1	0	2
House yard	9	17	1	0	11	25	63
Others	0	4	0	0	0	12	16
Total	100	100	104	100	99	98	601
Why does (Mr./Mrs.) dispose trash?							
Self-regulated effort to maintain cleanliness	80	81	82	94	68	62	467
Designated location	0	1	1	0	0	0	2
Required by law	20	4	8	5	5	0	42
Persuaded by others	0	0	11	1	2	0	13
Total	100	86	102	100	75	62	525
When did (Mr./Mrs.) first learn to correctly dispose waste?							
Before starting school	25	77	41	37	23	38	241
During schooling years	55	5	48	38	38	2	186
After finishing school	8	0	1	19	8	0	36
After starting family	12	4	9	6	2	23	56
Total	100	86	99	100	71	63	519
Was (Mr./Mrs.) taught not to litter?							
Yes	69	85	70	95	70	59	448
No	19	1	30	5	4	5	64
Total	88	86	100	100	74	64	512
If yes, through what channels was (Mr./Mrs.) taught not to litter?							
School	1	8	36	31	6	7	89
Peers	0	0	1	2	0	0	3
Peer Groups	0	0	1	15	3	0	19
Total	74	84	76	95	74	63	466

From Table 1, it can be observed that the Maluku Province has both the most income as well as expenditure per capita relative to other provinces. West Sumatra has the lowest expenditure per capita, followed by Jakarta. Citizens of Maluku, West Sumatra, and Jambi tend to be least concerned about their living environment (as indicated by the low number of households that suitably disposed garbage the last time they did). The most prevalent reasons why the inmates of some households disposed garbage correctly are self-initiative to maintain a clean environment or exemplary behavior for others to emulate. These desirable habits are most likely formed before these citizens entered school, as indicated by respondent answers on having such habits before beginning school. Additionally, more than 80% of them were taught not to litter during their schooling years. This is an indication of how investment through education influences not only human productivity in the labor market, but also the ability to make good choices [2], including those that affect investments in their wellbeing, as in the case of waste management. An understanding of good health can encourage individuals to follow precautionary behaviors such as maintaining a clean environment through responsible actions related to proper waste management. These choices are lacking for those with no or little knowledge of environmental cleanliness as the key to their health and wellbeing.

According to the summarized statistics described previously, there is a significant difference between the means of the per capita expenditure among provinces and cities. The significant differences among geographical factors show that the data are normally distributed across locations in terms of socioeconomic conditions.

The scores of the overall living conditions and living environments also differ significantly across the expenditure groups. A low score for the living conditions is observed in the case of low-expenditure households and regions with residents with low daily expenditure [3,4,5]. The significant differences among citizens grouped by expenditure per capita also show that the living conditions are normally distributed across income groups.

In the study, the first part of the analysis focused on detecting whether the self-assessment scores significantly differed among the groups of respondents. In addition, the analysis focused on detecting the existence of interactions between the tested variables [6,7].

The first stage involved the evaluation of the score of the overall living conditions. This score was measured on a scale of 1 to 5, with 1 denoting unsatisfactory and 5 denoting very satisfactory and comfortable. The score categorization was explained by the surveyors under certain criteria. From Table 2, it can be observed that this score differs significantly across cities and income groups. However, the geographical location and income-group categories exhibit no correlation; there is no interaction between these variables that affects the living-conditions score [8,9,10].

**Table 2:** Two-Way Analysis of Variance (ANOVA) Results (with Interactions) of Overall Living Conditions by Cities, Income Groups, and their Interactions.

Number of observations	506			R-squared	0.2716
Root MSE	0.710327			Adj R^2^	0.1916

Source	Partial SS	Df	MS	F	Prob > *F*

Model	85.600822	50	1.7120164	3.39	0.0000
Cities	27.299067	10	2.7299067	5.41	0.0000
Deciles of PCE	6.7022029	6	1.1170338	2.21	0.0407
Interactions	10.492612	34	0.30860625	0.61	0.9598
Residual	229.57704	455	0.50456493		
Total	315.17787	505	0.62411459		

The next stage involved the evaluation of the environmental hygiene/cleanliness score. Environmental cleanliness was measured from scale 1 to 5, with a score of 5 denoting the highest level of hygiene. The ANOVA test results in Table 3 indicate that there exist mean differences in environmental hygiene by cities and expenditure groups (as indicate by statistical significance). However, there are no significant interaction factors or tendencies attributable to living in certain cities, and citizens in certain income/expenditure groups exhibit significantly deteriorating or improving hygiene behaviors. In other words, while the score variable and the choice of city may be correlated, they appear as independent variables [11].

**Table 3:** Two Way Analysis of Variance (ANOVA) Results (with Interactions) of Environmental Hygiene Score, Income Groups, and their Interactions.

Number of observations	504			R-squared	0.2828
Root MSE	0.654463			Adj R^2^	0.2037

Source	Partial SS	Df	MS	F	Prob > *F*

Model	76.523707	50	1.5304741	3.57	0.0000
Cities	25.888047	10	2.5888047	6.04	0.0000
Deciles of PCE	5.6614766	6	0.94357943	2.20	0.0417
Interaction	14.981361	34	0.44062825	1.03	0.4266
Residual	194.02986	453	0.428322		
Total	270.55357	503	0.53787986		

As regards the score of the knowledge/awareness of the need for a healthy and clean environment (Table 4), the observed trend is similar to the previous two cases. A statistical significance of the mean differences is observed based on the city and income/expenditure. People living in better economic conditions are more knowledgeable. Furthermore, there is no interaction factor between the city of living and the score of environmental consciousness, which indicates that there are no tendencies of variable interactions that affect the score [3]. These two parameters may also be correlated; however, they appear as independent variables.

**Table 4:** Two Way Analysis of Variance (ANOVA) Results (with Interactions) of Environmental Consciousness Score by Cities, Income Groups, and their Interactions.

Number of observations	506			R-squared	0.3403
Root MSE	0.70732			Adj R^2^	0.2678

Source	Partial SS	Df	MS	F	Prob > *F*

Model	117.4219	50	2.3484376	4.69	0.0000
Cities	37.22526	10	3.7225262	7.44	0.0000
Deciles of PCE	6.348402	6	1.058067	2.11	0.0504
Interactions	25.61437	34	0.7533637	1.51	0.0363
Residual	227.6374	455	0.50030199		
Total	345.0593	505	0.68328572		

As regards the score of environmental cleanliness/hygiene (Table 5), the results are similar to those of the previous cases. There is a statistical significance of the mean differences based on cities and income/expenditure. People living in poorer economic conditions tend to live in less cleaner environments. However, there is no interaction between the city of living and the cleanliness score; there are no tendencies of variable interactions that affect the score [12]. These two variables again appear independent of each other.

**Table 5:** Two Way Analysis of Variance (ANOVA) Results (with Interactions) of Environmental Cleanliness by Cities, Income Groups, and their Interactions.

Number of observations	494			R-squared	0.1627
Root MSE	0.863457			Adj R^2^	0.0681

Source	Partial SS	Df	MS	F	Prob > *F*

Model	64.16	50	1.28	1.72	0.0024
Cities	17.97	10	1.80	2.41	0.0085
Deciles of PCE	12.76	6	2.13	2.85	0.0098
Interactions	23.46	34	0.69	0.93	0.5918
Residual	330.28	443	0.75		
Total	394.44	493	0.80		

The last stage of the analysis focuses on how the chosen variables affect each other and the significance of the correlation. The confidence level in the following analysis was set to 95%, with the alpha value (significance level) set to 5%. Table 6 summarizes the Spearman correlation matrix.

**Table 6:** Spearman Coefficient Correlation Matrix.

	Overall Living Conditions	Overall Environmental Cleanliness	Knowledge of Environmental Cleanliness	Personal hygiene/Cleanliness Lifestyle	Littering Behavior	Expenditure/Capita	Expenditure/Capita (Decile)	Income/Capita (Decile)
Overall Living Conditions	*1*							
						
Overall Environmental Cleanliness	0.4015*	*1*						
	0							
Knowledge of Environmental Cleanliness	0.5318*	0.5267*	*1*					
	0	0						
Personal Cleanliness Lifestyle	−0.4175*	−0.3643*	−0.5066*	*1*				
	0	0	0					
Littering Behavior	0.1273*	0.02	0.1560*	−0.0389	*1*			
	0.0067	0.6713	0.0009	0.4096				
Expenditure/Capita	0.1901*	0.1535*	0.1313*	−0.0844	−0.0592	*1*		
	0	0.0011	0.0052	0.0732	0.2088			
Expenditure/Capita (Decile)	0.2144*	0.1662*	0.1588*	−0.1030*	−0.0289	0.9732*	*1*	
	0	0.0004	0.0007	0.0285	0.5402	0		
Income/Capita (decile)	0.1719*	0.1204*	0.1325*	−0.0655	−0.0133	0.6169*	0.5898*	*1*
	0.0002	0.0104	0.0048	0.1645	0.7786	0	0	

In Table 6, the coefficient correlation is indicated by enlarged numbers in gray boxes, and the numbers in bold (accompanied by an asterisk) indicate strong and significant correlations. The variables in blue-colored fonts indicate that their values were acquired via self-assessment of respondents [2].

The abovementioned findings are convergent with those of other studies [13,14,15]; these studies also reported that a clean and healthy lifestyle in conjunction with education generally correlated with clean environmental conditions. A clean and healthy lifestyle is not usually replaced by an unhygienic lifestyle. In addition, households greater economic prosperity (indicated by a positive correlation with expenditure or income per capita) usually have better living conditions. Better economic conditions, in this case, also aid people in keeping the environment clean. This finding indicates why slums often appear unkempt and unhygienic [5]. A surprising finding refers to the dummy variable of littering behavior. A positive correlation between the overall living conditions and littering behavior indicates that households with littering behavior also tend to score higher on the living conditions. This significant positive relationship is an indicator of self-interest and ignorance. In general, social behavior and lifestyle should suitably conform with government policies [16]. This trend also shows the need for participation in waste management at the community level [17].

The major factor toward creating a clean and healthy lifestyle among urban citizens is environmental consciousness, as indicated by the coefficient correlation of 0.526, meaning that the overall environmental cleanliness score will increase by 0.526 when the environmental consciousness score is increased by 1. Here, it is noted that such a correlation is not an accurate reflection of how urban citizens handle waste; however, it reveals how each of the variables correlates and the anomalies occurring in society.

## Experimental design, materials, and methods

The experimental design also aimed at evaluating how economic conditions correlate with the littering behavior of the households surveyed. The analysis also measured the significance of the mean differences among groups based on the survey data. Here, it is noted that the survey covered 600 households from 6 different provinces, with 100 samples being acquired from different cities within these provinces. Importantly, the respondents were residents and not migrants. The dataset containing the respondent records was suitably filtered by data processing software. In particular, qualitative records were encoded, and quantitative ones were cleared from string inputs.

The survey also elaborates the socioeconomic dimensions of waste-handling behavior by focusing on the economic status, which affects the quality of living and waste-handling behavior of urban citizens. These indicators were embedded in the questionnaire form, which consisted of eight sets of questions. The details and description of the survey are listed in Table 7. The respondent economic conditions were measured via the approximate expenditure and income for each household. The income and expenditure were stated in Rupiah, and they were converted to USD assuming that 1 USD equals IDR 14,071 (average exchange rate in October 2019). The approximate income and expenditure per capita were calculated by dividing the approximate household income and expenditure by the number of household members.

**Table 7:** List of survey sessions.

No.	Sessions	Description of Sessions	Observation Level	Types of Questions/ Responses
1	Description of Spatial and Household Recognition	This part of the questionnaire records the respondent's household and individual ID along with their geographical location (province, city, district, etc.).	Household	Short Answers
2	Summary of Household Economic Conditions	This part of the questionnaire records the household income and expenditure per day, number of household members, and possession of social insurances.	Household	Short Answers
3	Description of Household Members	This part of the questionnaire records each household member's age, education, gender, marital status, and working status.	Individual	Short Answers, Multiple Choices
4	Description of Self-Regulation with Regard to Disposing Garbage	This part of the questionnaire records individual waste-handling behaviors (where to usually place trash, etc.) and reasons behind personal waste-handling habits.	Individual	Multiple choices
5	Description of Knowledge and Contribution to Waste Handling	This part of the questionnaire records the knowledge level of waste handling, personal waste management habits, and individual contribution to waste-handling activities.	Individual	Scoring and Checklists
6	Description of Perception and Attitudes on Waste Handling	This part of the questionnaire records individual perceptions and reactions as regards correct and incorrect waste handling. Furthermore, this session also records personal responses to local waste problems.	Individual	Multiple Choices, Scoring, and Checklists
7	Description of Guided Self-Assessment Conditions	This part of the questionnaire records guided self-assessment scores on living conditions, living state, knowledge of environment, environmental cleanness, lifestyle, and personal satisfaction. Scores in this session are based on guidance by surveyors.	Individual	Scoring

The following variables were scored based on self-assessment (from session 7): (i) living state/conditions, (ii) knowledge/awareness of healthy environment and lifestyle, (iii) cleanliness and hygienic living conditions, and (iv) hygiene and lifestyle relative to socioeconomic conditions (approximated by decile group of expenditure per capita), city of living, and the dummy variable of littering behavior. The self-assessment scores are designed such that higher scores correspond to better conditions and *vice versa*. The self-assessment scores were not completely subjective as the surveyor explained the classification criteria for each score to the respondents. Hence, the scores can be considered as guided self-assessment scores.

The variables were analyzed via various possible analysis of variance (ANOVA) approaches in conjunction with the non-parametric Spearman's correlation test. The tests were conducted to check whether these variables exhibit a significant mean among the categories of cities of living, decile group of incomes, and households with littering habits.

In addition, the Spearman's rank correlation matrix (including significance levels and asterisk) was used to analyze the correlation tendencies between variables. The significance levels and asterisk under the coefficient correlation mark important significant tendencies between two variables, and this can enable focus on the factors of interest. The recorded significance levels can also guide researchers and policymakers on what factors or variables they should focus on to raise awareness of the need for a clean and healthy lifestyle.

In inferential statistical analysis, the definitions of groups (categories) or the division of respondent groups are important. In this study, respondents (households) were classified into several types of groups: First, the domicile-based group categorized respondents at the city or province level. Second, respondents were categorized based on waste-handling behaviors or littering. Here, it must be noted that only some households were surveyed on their littering behavior. Thirdly, respondent data were classified into deciles based on their expenditure per capita. The 1st decile indicated the 10% of respondents with the lowest expenditure per capita, while the 10th decile indicated the 10% of respondents with the highest expenditure per capita. For the sake of simplicity, the summarized statistics are provided based on domicile-based groups at the province level.

## Declaration of Competing Interest

The authors declare that they have no known competing financial interests or personal relationships which have, or could be perceived to have, influenced the work reported in this article.

## Appendix Supplementary materials

Image, application 1

Image, application 2

Image, application 3
