# Supplementary material for: Dataset on waste management behaviors of urban citizens in large cities of Indonesia
Source: Data Brief. 2020 Jul 22;32:106053. doi: 10.1016/j.dib.2020.106053 (PMC7394850; doi:10.1016/j.dib.2020.106053)
Supplement: Supplementary file 3 [file mmc3.pdf]

Language Editing Services

Registered Office:
Elsevier Ltd
The Boulevard, Langford Lane,
Kidlington, OX5 1GB, UK.
Registration No. 331566771

To whom it may concern

The paper "Dataset on Waste Management Behaviors of Urban Citizens of Big Cities in Indonesia" by Agus Brotosusilo was edited by Elsevier Language Editing Services.

Kind regards,

Elsevier Webshop Support

(This is a computer generated advice and does not require any signature)
